# The Effect of Sputtering Parameters and Doping of Copper on Surface Free Energy and Magnetic Properties of Iron and Iron Nitride Nano Thin Films on Polymer Substrate

**DOI:** 10.3390/ma10020217

**Published:** 2017-02-22

**Authors:** Waheed Khan, Qun Wang, Xin Jin, Tangfeng Feng

**Affiliations:** College of Materials Science and Engineering, Beijing University of Technology, Beijing 100124, China; jinxin1118@emails.bjut.edu.cn (X.J.); ftfeng1993@emails.bjut.edu.cn (T.F.)

**Keywords:** iron nitride, functional properties, surface free energy, EMI shielding effect, magnetron sputtering, polymer substrate

## Abstract

The objective of this study was to deposit thin films on PET polymer substrate and examine the functional properties systematically. Their properties have been studied as a function of the N_2_-Ar flow rates, deposition time span and Cu doping. Iron nitride film deposited on both sides exhibits ferromagnetic phases, γ′-Fe_4_N and ε-Fe_3_N co-existed, shows negligible magnetic anisotropy. Other samples show the evolution of N-rich (FeN, Fe_2_N) and N-poor (Fe_16_N_2_, Fe_3_N, Fe_4_N) phases under different deposition time conditions. XPS analysis and free energy calculations confirmed that co-sputtered Fe-Cu thin films are more stable than layer deposited counterparts. From VSM results it is evident that the dominant phase, changes steadily from the ferromagnetic α-Fe (N) to the paramagnetic ξ-Fe_2_N with the increase of nitrogen flow rates and the ordering of the nitrogen atoms. Binding energy increases steadily from 733 eV to 740 eV with the increasing thickness of thin films from 74 nm to 94 nm. It was observed that surface energy decreases as the contact angle of glycol increases and changes the thin film surface from polar to nonpolar. TEM images indicate that cubic γ′-Fe_4_N and ε-Fe_3_N nano particles oriented in preferred directions dispersed uniformly in the amorphous iron nitride matrix.

## 1. Introduction

Soft magnetic Iron nitride thin films have appealed much consideration and are strongly desired for film transformers in integrated circuits and high-frequency applications [1,2]. The magnetic iron nitrides (N at % < 25) are chemically inert and have mechanically hard surfaces [3]. This, adding with the intrinsic magnetic properties, makes them a suitable material in devices such as recording heads being a better alternative to pure iron [4].

In the Fe-N binary phase diagram the major phases are Fe_16_N_2_, Fe_4_N, Fe_3_N, Fe_2_N, FeN, and Fe_3_N_4_ with an increasing atomic percentage of N [5]. Compound forming at. 11 at % N is α″-Fe_16_N_2_ have a giant magnetic moment [6]. The γ′-Fe_4_N phase is formed around 20 at % N which has distinct magnetic properties and FCC crystal structure [7] and this phase can also be produced by controlled annealing of FeN and accordingly provides a cause of spin injection for semiconductors [8]. Ferromagnetic Fe_3_N phase changes to paramagnetic Fe_2_N at room temperature as the N at % increases from 25 to 33. In the last decade or so, paramagnetic ξ-Fe_2_N and nonmagnetic iron nitride phases also have received ample attention [9]. With a magnetization of 72.346 and the coercivity of 85.54 Oe ε-Fe_3_N can be useful in several applications [10].

Thin films possess high surface-to-volume ratio which plays an important role in the polar and dispersion contributions to the solid surface energy and can be determined in accordance with Young’s equation [11], Fowkes theory [12], and Owen and Wendt theory [13]. The unbalance forces for the molecules at the surface leads to additional energy at the surface, and this additional energy at the surface is known as surface free energy which depends mainly on chemistry of the surface, and significantly can be controlled by synthesis parameters [14]. The surface energy was calculated for various thin film systems according to the measured contact angles [15,16,17]. The outcomes show that the surface energy of NiFe films is inversely proportional to the crystallinity and grain size [18]. No significant work has been done yet on measuring the surface free energy of iron nitride thin films.

Mostly previous research has been done to grow iron nitride thin films on the Si (110), Si (100), corning glass, MgO (100) and quartz glass non-flexible substrates. These substrates are incompatible for certain applications where flexibility, light weight and toughness are needed. In order to overcome these shortcomings, flexible substrates, which are made of plastic, have been used instead of glass. Until now, no systematic investigation has been published about synthesis and properties of FeN thin films on flexible substrates. Although Indium tin oxide (ITO) thin films have been deposited on flexible polymer substrates to evaluate the optoelectronic properties used in filters for plasma displays, low-e windows, solar cells, etc. [19,20,21,22].

Magnetic, structural and other functional properties of FeN thin films can be tuned by controlling the sputtering parameters. The magnetic anisotropy of the iron nitride thin films can be altered by changing the oblique sputtering angle and the deposition time. The IPUMA increases with the increase of oblique angle [1]. Dissimilar phases of FeN films can be achieved through changing N_2_ partial pressure in a mixture of N2/Ar gas flow and the surface becomes smoother as N_2_ fraction increases due to different growth conditions [23]. The nucleation of γ′-Fe_4_N can be facilitated by the addition of hydrogen gas in the flow gas mixture during sputtering [24]. The coercivity of the films increases directly with substrate temperature and decreases with the increase of substrate bias voltage [5]. Coercivity of the films increases and the magnetic moment of the Fe atoms decreases almost linearly with the nitrogen content [25].

Thermal stability of FeN compounds is low due to the weak Fe-N bonding which results in low magnetic properties. This problem can be solved by the doping of third element/s. Magnetic properties of the films enhanced markedly with Al addition, however Zr addition has only a minimal effect on the thermal stability of FeN thin films [26]. Akhil et al. reported that addition of 12 at % Al results in the presence of Al atoms at grain boundaries and dissolved substitutionally improves the thermal stability without any change in grain size [27].

The main objectives of this work are, to develop iron nitride nano thin films on flexible PET substrate, to study the effect of processing parameters on the magnetic and surface properties of iron nitride polycrystalline thin films fabricated by DC reactive magnetron sputtering. We also prepared FeN thin films doped with copper as a second layer and co-sputtering of Fe and Cu targets was done to prepare nano thin films. Additionally, a unique two sided sputtered film of FeN was prepared which is a potential candidate for twin sided view panels with operative functional properties.

## 2. Experimental Details

Granular iron nitride and copper doped iron thin films were prepared by DC reactive magnetron sputtering on flexible PET substrates. Polymer substrates were provided by lucky corporation, China previously printed with pure silver layer of 4–6 nm thick. For deposition of iron nitride thin films, a 75 mm diameter iron target (99.995% pure) in the 5n pure gas mixture of N_2_ and Ar was used with varied gas mixtures and flow rates while keeping the substrate at room temperature. The Ar and N_2_ flow rates were precisely controlled by a mass flow controller. A base pressure of about 6.6 × 10^−5^ Pa was attained prior to the deposition. During the deposition, aggregate partial pressure of Ar + N_2_ mixture was 0.55 Pa and the sputtering power was kept constant at 115 W. The distance between the target and the substrate was about 100 mm. In case of Cu doping and co-sputtering of iron and copper, 99.995% pure Cu target of 75 mm diameter and 2 mm thickness was used. To increase homogeneity, the substrate holder was rotated at 8 rpm.

**Figure 1:**
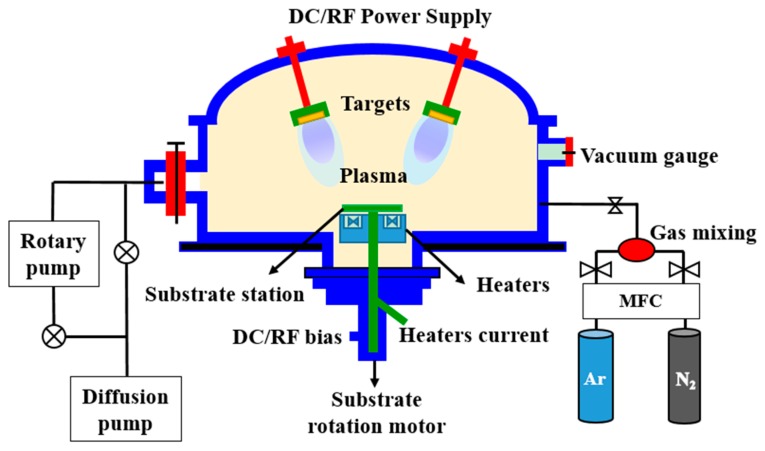
The schematics of dual power (DC/RF) multi target magnetron sputtering system used.

Additionally, a unique double sided nano thin film of FeN was deposited. The schematic of experimental facility is shown in Figure 1. The sample ID’s and processing parameters are given in Table 1. The thickness of the films was measured by a surface profilometer (VEECO Dektak8 ADP-8, BJUT, Beijing, China).

The structures of the films were examined using X-ray diffraction (XRD) with Cu Kα radiation (λ = 0.15406 nm) using a current of 30 mA and voltage of 40 kV (Shimadzu, XRD 7000, BJUT, Beijing, China). Continuous scan was acquired with drive axis = θ–2θ in the range of 20° to 80°. The in-plane static magnetic properties of thin films were measured using vibrating sample magnetometer equipped with MicroSense easy VSM software 9.13 WA in magnetic fields up to 2.1 T. Thermo ScientificNational Nanotechnology Centre, Beijing, China ESCALAB 250x, X-ray photoelectron spectroscopy (XPS) (Chinese academy of Sciences, Beijing, China) was used to determine the chemical binding of atoms and composition. Monochromatic source of Al Kα radiation with energy resolution of 0.5 eV and voltage approaching to 1500 eV was applied.

Contact angle measurements were conducted with a “Drop Shape Analysis System” (Kruess DSA100, National Nanotechnology center, Beijing, China) at ambient temperature (25 ± 2 °C) and relative humidity (30% ± 5%). Volume of liquid droplet was 4 μL and carefully deposited onto the sample surface with a dosing rate of 2 μL/s. The surface energies were calculated from the contact angles [15,16,17] of pure test liquids water and ethylene glycol (C_2_H_6_O_2_). Calculations were based on 3–5 repeats in each sample and averaged for more precise results. The power absorption EMI shielding effect in the range of 10 MHz–3.0 GHz (Bluetooth range) was analyzed by means of AV36580A Vector network analyzer using a micro-strip line with the sample placed in the middle of the strip line. The microstructures of the films were examined by a FEI Tecnai G2 F30 transmission electron microscope (TEM) operating at 200 kV. Samples for TEM observation were bonded with epoxy resin and then cut by using a diamond knife on an ultra-microtome. 3 mm dia sample discs were around 100 nm thick and remaining traces of resin were removed by cleaning in acetone. All the above measurements on thin films were accomplished at room temperature.

## 3. Results and Discussion

### 3.1. X-ray Diffraction Structural Properties

**Figure 2:**
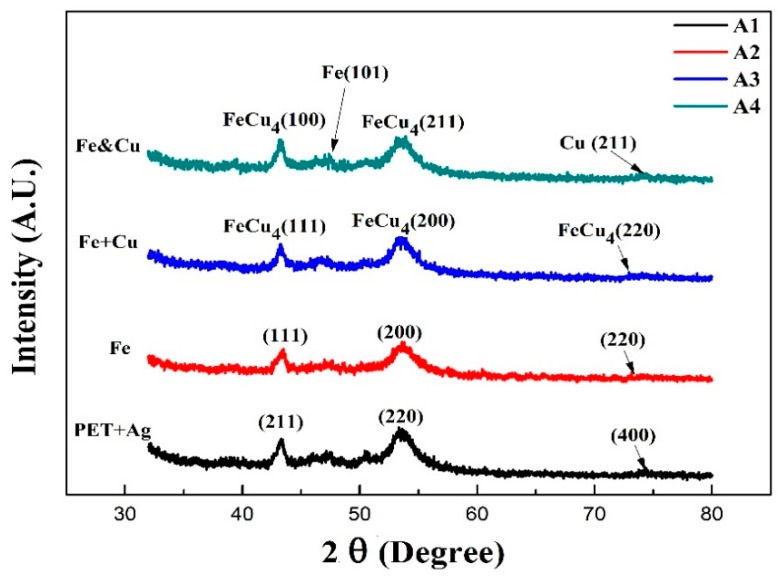
XRD pattern of the Cu-dopped nano structured iron thin films.

Figure 2 shows the XRD pattern of pure iron and copper doped iron thin films. A1 is the as received PET polymer sample with a thin layer of Ag printed for better adhesion properties during magnetron sputtering. Evident diffraction peaks of pure silver crystal faces are at 43° (211), 55° (220) and 74° (400), JCPDS # 070-1019. Sample A2 is the pure iron thin film, results indicate the presence of two peaks at 42.9° and 73.35° corresponding to (111) and (220) planes of paramagnetic γ-Fe crystal structures, while the peak at 49.96°correspond to (200) plane of ferromagnetic α-Fe (JCPDS #065-4150).

Double layered thin film of sample A3 was deposited in two steps, firstly the pure iron was sputtered for ten minutes and then doping with copper was achieved at the same sputtering power for five minutes. The diffraction peaks at 2θ: 43.27°, 50.39° and 74.05° corresponds to the (FCC) phase of FeCu_4_ (111), (200) and (220) in XRD patterns of JCPDS #065-7002 with a lattice parameter a = 3.6184 Å. Thin film of sample A4 was co-sputtered with Fe and Cu simultaneously for 15 min, minor XRD peaks of Fe (101) and Cu (211) are depicted. This reveals that a trivial amount of Fe and Cu remains pure due to insufficient time for reaction in the plasma atmosphere. The other evident peaks of FeCu_4_ phase are same as in sample A3 but with a slightly higher intensity and at different crystal orientations of (100) and (211). The difference in orientation implicates the presence of favorably oriented grains that can assist in the evolution of surface free energy and static magnetic properties as explained later.

**Figure 3:**
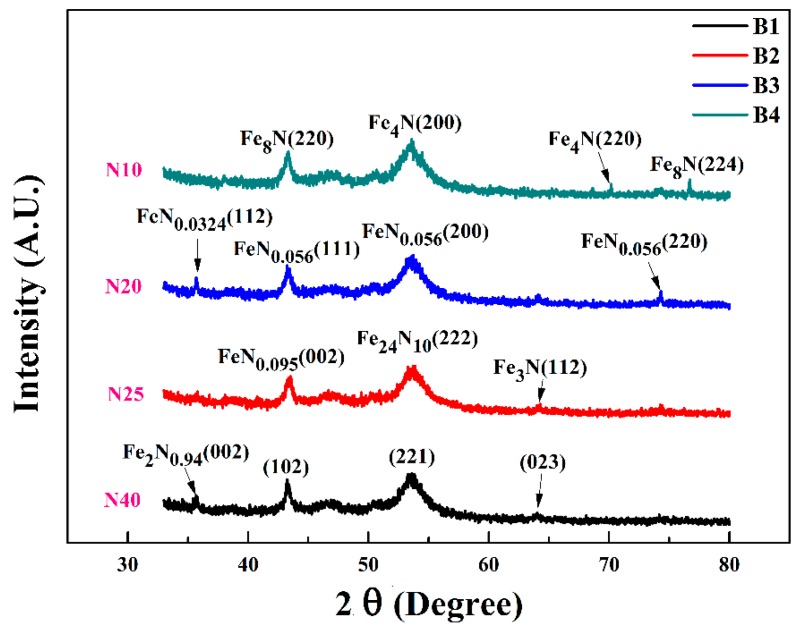
The XRD pattern for iron nitride films grown at different nitrogen flow rates.

Figure 3 demonstrates the XRD patterns of FeN films deposited on PET substrates with different nitrogen flow rates. The gas mixture in the sputtering process is the most important deposition parameter, and has a significant influence on the composition and the phase structure of the films. Many properties of thin films depend on the sputtering pressure [28]. Varying flow rates of nitrogen from 10 to 40 sccm resulted in nitrogen-poor to nitrogen-rich crystalline phases of FeN along with amorphous matrix [29].

At a higher flow rate of nitrogen, Sample B1 shows the major peaks of crystalline Fe_2_N_0.94_ at 42.5° and 56.377°. While the characteristic peaks at 67° and 75.4° are not evident indicating the amorphous nature of matrix (JCPDS#086-1025). With decreasing nitrogen flow rates, the magnetic phases ε-Fe_2.4_N and FeN_0.056_ appears in sample B2 and B3 respectively. Fe_2.4_N (222) have lattice constant a = 3.215 and FeN_0.056_ (200) have lattice constant a = 3.6 with a slight difference in the density from 7.69 g/cm^3^ to 8 g/cm^3^ respectively. When nitrogen flow decreased to 10 sccm, the main cubic phase of γ″-Fe_4_N appears. The lattice parameters for γ″-Fe_4_N is a = 3.795 Å and density is 7.24 g/cm^3^ which is comparable to that of Jacobs et al. [30]. In sample B1, the strong peak of α″Fe_8_N (220) at 44.78° and a weak peak (224) at 76.7° are seen, having space group#139 with a lattice constant of 5.72 Å. To summarize, with the decrease of N_2_ flow rate from 40 to 10 sccm; the phase evolution can be designated as:

Fe_2_N_0.94_ => FeN_0.095_ => Fe_2.4_N => FeN_0.056_ => Fe_4_N => Fe_8_N.

**Figure 4:**
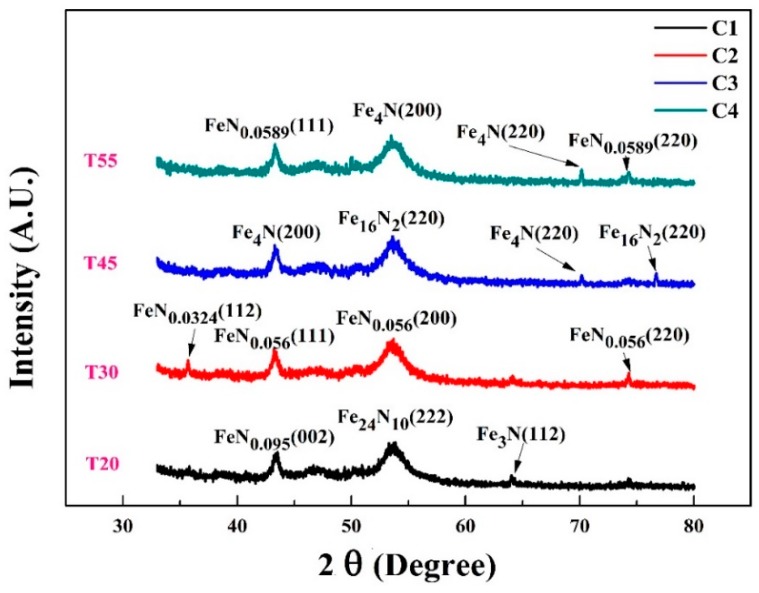
The XRD pattern for iron nitride films grown at different deposition time spans.

Figure 4 displays the XRD patterns of FeN films deposited on PET substrates at varying deposition time spans from 20 min to 55 min while keeping the nitrogen flow constant at 10 sccm. A logical evolution of iron nitride phases can simply be acquired by changing the time of nitrogen reactivity to occupy interstitial sites within the bcc-Fe lattice [26]. For the samples with sputtering time of 20 min (C1), 30 min (C2), 45 min (C3), and 55 min (C4), their thickness is 74 nm, 79 nm, 94 nm and 184 nm respectively as shown in Table 1. The sputter yield increased with increase of deposition time, resulting in the increase of Fe atoms in nitrogen poor phases. In the instance of 184 nm thick sample (as shown in Figure 4(C4)), except for a weak peak at 70.18° resulting from γ′-Fe_4_N (220), there is a strong peak at 54° corresponding to γ″-Fe_4_N with preferred orientation at (200). With decreasing deposition time, concentration of iron and nitrogen atoms become lesser hence thickness of the films decreases. The evident peaks as shown in Figure 4(C3), Figure 4(C2) and Figure 4(C1), corresponds to Fe_16_N_2_, FeN_0.056_ and Fe_24_N_10_ at the preferred orientations (220), (200) and (222) respectively.

**Figure 5:**
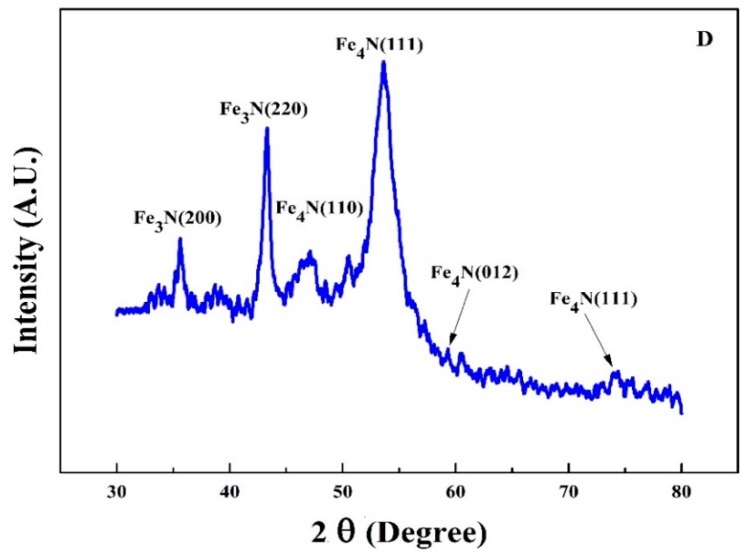
The XRD pattern of sample D the iron nitride thin film deposited on both sides of PET.

Figure 5 shows the XRD pattern of FeN (sample D) deposited on both sides of PET substrate under a slightly increased Ar flow rate of 50 sccm. As the magnetic properties of iron nitride are closely associated to the crystallographic phases, the ability to produce as many pure phases as possible is highly appropriate for their applications in magnetic functional devices [31,32]. Two nitrogen-poor ferromagnetic phases, γ′-Fe_4_N and ε-Fe_3_N co-exist in this thin film validates that the magnetic moment of an Fe atom in iron nitride is strongly influenced by its nearest N neighbors, these phases have excellent thermal stability and large spin polarizations [33]. The strong peak of γ′-Fe_4_N with preferred orientation along (111) direction is evident at 53° having density of 2.2 g/cm^3^, JCPDS#77-2006. While the strongest peak of ε-Fe_3_N (220) is shown at 43.35°, it belongs to metallic ferromagnetic phase at room temperature with Curie temperature of about 300 °K, JCPDS#73-2101. The diffraction peaks for ε-Fe_3_N move monotonically in the direction of smaller angles, which proposes that the increased nitrogen concentration expands its unit cell.

### 3.2. Magnetic Properties

**Figure 6:**
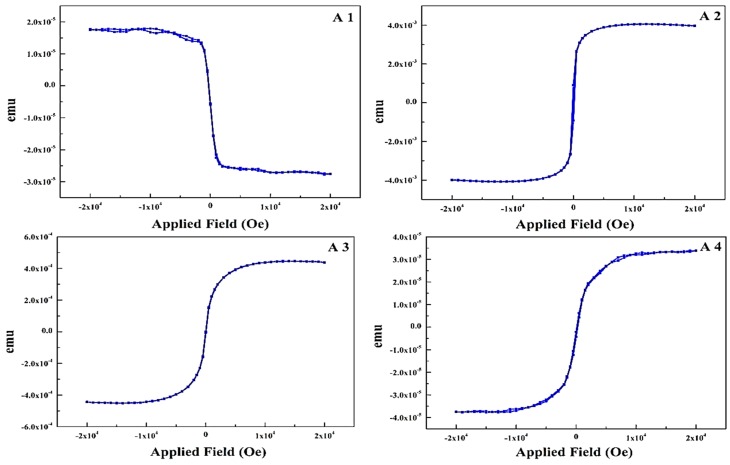
Magnetization measurements for the sample A1, A2, A3 and A4 respectively. (**A1**) The hysteresis loop for the pure PET with Ag printing; (**A2**) the hysteresis loop for pure Fe; (**A3**) the hysteresis loop for Fe + Cu and (**A4**) the hysteresis loop for co-sputtered Fe and Cu.

The in-plane static magnetic properties of thin films were measured using vibrating sample magnetometer, as shown in Figure 6A1–A4. Easy axes of all the samples are in the film plane because of shape anisotropy.

A1 is the hysteresis loop of as-received PET sample with a layer of 3–5 nm printed Ag, shows the inherent diamagnetic nature of both PET and Ag. This a quantum mechanical effect that occurs in Ag and creates an opposing magnetic field with susceptibility equals to −2.1 × 10^−5^. A2 is the 33 nm thick pure Fe thin film contributing to a saturation magnetization (Ms) of 3.81 × 10^−3^ emu. The lower value of Ms is due to the PET substrate and presence of Ag layer. It is clear that the diamagnetic nature of sample changes to ferromagnetic with a very low remnant magnetization (Mr) of 913.96 × 10^−6^ emu at H = 0. Sample A3 is the hysteresis loop of a 5 nm thick layer of pure Cu on top of Fe, it shows slightly lower Ms (4.21 × 10^−4^ emu) as compared to sample A2 because of the Cu layer. These structures are of significant concern since ferromagnetic and antiferromagnetic phases are very sensitive to the lattice parameter and symmetry due to different magnetic moments [34]. A4 is the hysteresis loop of co-sputtered nano thin film of Fe and Cu having thickness equals to 34 nm. The Ms is nearly same as in sample A3 with Mr of 2.4 × 10^−6^ emu, these magnetic properties are influenced by the Fe thickness and film-growth parameters. Meanwhile nanoscale FCC Fe is exposed to strain and lattice distortions that can alleviate diverse magnetic states, the magnetic moment of Fe is very sensitive to the environment unlike Ni and Co [35], and can be stable at temperatures either as coherent precipitates in Cu or metastable FeCu alloys [36]. Nitrogen-poor phases (Fe_4_N, Fe_3_N and Fe_16_N_2_ etc.) exhibit a high magnetic moment. Amorphous or nanocrystalline phase of FeN can be achieved at low nitrogen pressure and flow rates [37,38].

**Figure 7:**
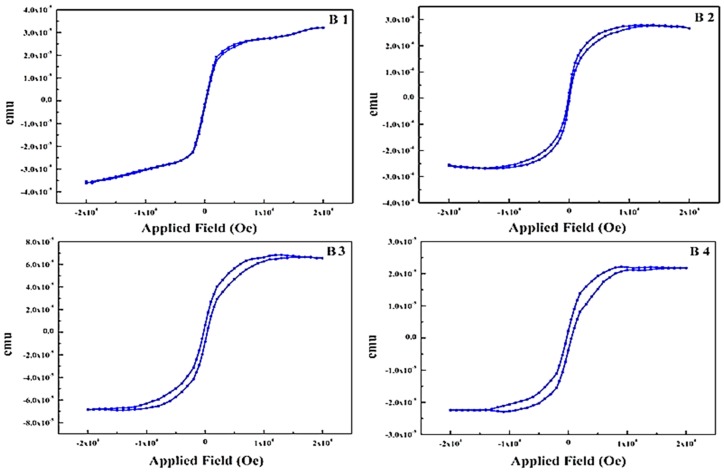
Magnetization measurements for FeN thin films at varying flow rates of N_2_. (**B1**) N_2_ flow rate = 40 sccm; (**B2**) N_2_ flow rate = 25 sccm; (**B3**) N_2_ flow rate = 20 sccm; and (**B4**) N_2_ flow rate = 10 sccm.

However, a systematic and detailed study of the phase evolution of nanocrystalline iron nitrides phases on a polymer substrate was not yet conducted. Figure 7 (B1, B2, B3 and B4) shows the dependence of saturation magnetization (Ms) and the coercive force (Hc) at decreasing nitrogen flow rate. At a higher flow rate of nitrogen, 40 sccm, sample B1 exhibits Ms exceeding to 3.5 × 10^−5^ emu and it is hard to reach a saturation point.

While at the decreased rates of 25, 20 and 10 sccm samples B2, B3 and B4 have a well-defined saturation point. The dependency of Ms on N_2_ flow can be clarified by the phase evolution revealed in Figure 3. As we know, α-Fe (N) and α″-Fe_16_N_2_ have high ranges of Ms. In contrast, the Ms of γ′-Fe_4_N and ε-Fe_x_N are lower and ξ-Fe_2_N is paramagnetic at room temperature. From Figure 3, it is evident that the dominant phase changes from the ferromagnetic α-Fe (N) to the paramagnetic ξ-Fe_2_N steadily with increase of the nitrogen flow rates and the ordering [39] of the nitrogen atoms.

**Figure 8:**
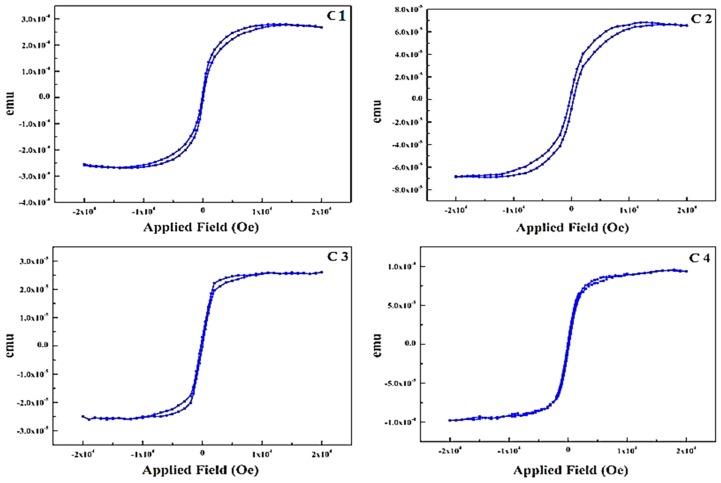
Magnetization measurements for FeN thin films at varying deposition time span and fixed flow rates of N_2_ = 10 sccm (**C1**) 20 min; (**C2**) 30 min; (**C3**) 45 min; (**C4**) 55 min.

The static magnetic properties of the iron nitride thin films with different thicknesses achieved under varying deposition time spans (t) were investigated by the in-plane hysteresis loop while keeping the nitrogen flow rate constant at 10 sccm. In Figure 8C1–C3, the magnetic hysteresis loops reveal that the iron nitride thin films show excellent soft magnetic properties at an approaching deposition time of 45 min and thickness around 94 nm. The saturation magnetization of sample C1 is 1.35 × 10^−3^ emu having thickness of 74 nm. With slight increase of film thickness in sample C2 the MS remains almost same, while the sample C3 (94 nm) and C4 (184 nm) exhibits an increased Ms = 1.6 × 10^−3^ emu and 1.54 × 10^−3^ respectively. This is a comparatively low value, which may as a result of the smaller particle size of sample, the dependence of saturation magnetization on grain size was proposed by Alben et al. [40].

(1) $$M_{s}A^{3}\;=\;0.64\left(\frac{{K_{1}}^{4}D^{6}}{H_{c}}\right)$$

When the average size D of magnetic particles is reduced, the interchange coupling among the magnetic particles may occur, which forces the magnetizations of particles to arrange in a parallel line, leading to a cancellation of magnetic anisotropy of individual particles and incapacitating the demagnetization effect. As a result, the average coercivity H_c_ of the film decreased and hence the Ms increased.

**Figure 9:**
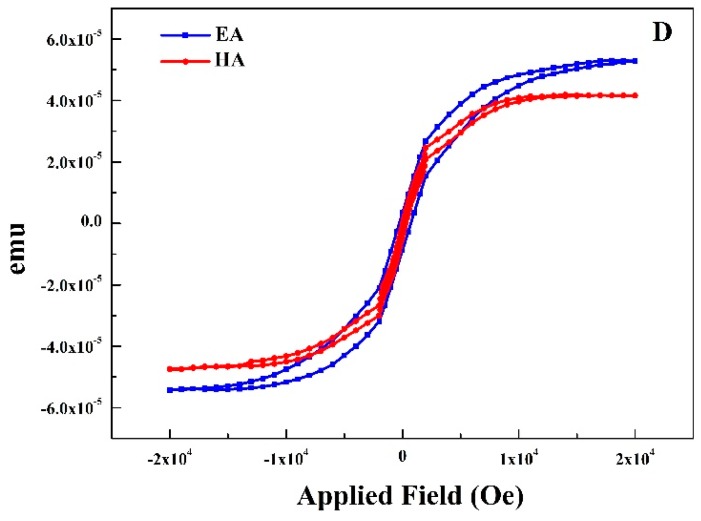
Magnetization measurements of the FeN thin film sputtered on both sides.

Magnetic properties of a specially developed thin film are shown in Figure 9, FeN is deposited on both sides under a mixture of Ar 50 sccm and N_2_ 10 sccm. It is evident that the Ms along easy axis and hard axis is almost same depicting negligible anisotropy. Uniaxial anisotropy of soft magnetic thin films is very important for high-frequency applications according to the following equation [41]

(2) $$f_{r}\;=\;\left(\frac{\gamma }{2\pi }\right)\sqrt{H_{k}}(4\pi M_{s}\;+\;H_{k})$$

where H_k_ is the anisotropy field and 4πMs is the saturation magnetization which can be adjusted by fine-tuning the magnitude of the in-plane uniaxial magnetic anisotropy. For one sided sputtered films, it always has been difficult to tailor those properties effectively. Several studies have been conducted before, including ex situ annealing in a magnetic field [41] and changing oblique angles [42] etc. We propose a simple scientific solution to address this and successfully controlled the uniaxial anisotropy by sputtering on both sides of a polymer substrate. When applied field is near zero (Figure 8) both easy axis and hard axis in M–H hysteresis loop shows a slop. It reveals that the sample consists of at least two magnetic phases which is in agreement with the result of XRD.

### 3.3. Binding Energy and Film Composition

**Figure 10:**
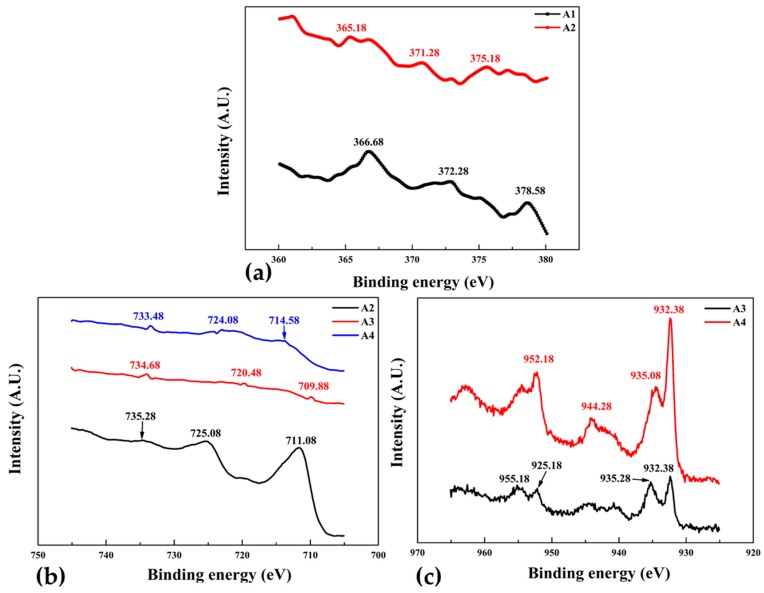
X-ray photoelectron spectra of group 1 thin films (**a**) Ag_3_d; (**b**) Fe_2_P; and (**c**) Cu_2_P.

X-ray photoelectron spectroscopy (XPS) is a surface-sensitive quantitative spectroscopic method that measures binding energy and analyze the elemental composition of a sample. The electron binding energy of individually emitted electrons can be calculated by using the Ernest Rutherford (1914) equation, as the energy of an X-ray with particular wavelength is known (Al K_α_ X-rays, *E*_p_ = 1486.7 eV). The wide scan of all the samples reveals that a very small O 1s content exists. Figure 10a shows the XPS spectra of samples A1 and A2 was performed to analyze the shift and change in intensities of Ag3d. The binding energy of silver in both samples increases consistently with the decrease of intensity. Different binding energies represents the different electronic states of Ag atoms, for instance the Ag printed PET sample A1 binding energy of 378.58 eV is related to the stable Ag^+^.

AS sample A2 was sputtered with pure Fe, the binding energy of 375.18 eV belongs to the much less stable Ag^2+^. Stability of silver can be attributed to the presence of Fe atoms in nearby locations, as the electronegativity of Ag and Fe are much different it is inferred that the change of electron distribution at the interface is due to Fe deposition. Overall the shift in binding energy is towards lower side as compared with the A1 sample which may be due to the Fe-Ag bond formation. Fe_2_p spectra of sample A2 shown in Figure 10b indicates the binding energy of 735.28, 725.08 and 711.08 eV related to ferromagnetic phases of Fe (111), (200) and (220) respectively which is in-agreement with XRD results shown in Figure 2, thus the ferromagnetic phases are somewhat favored over diamagnetic phase [43].

XPS spectra of sample A3 (Fe + Cu layered) and A4 (Fe-Cu co-sputtered) samples are shown in Figure 10b,c respectively. When two higher intensity Cu_2_p peaks of these samples are compared, binding energy at 955.18 (Cu^2+^) and 935.28 (Cu^+^) eV of sample A3 are slightly higher but intensities are considerably lower than the sample A4. It also reveals that the surface active components generally occur in the form of Cu^2+^ species. The Fe2P spectra, Figure 10b shows that the binding energy and intensities of sample A4 is greater than the sample A3. Noticeable component of the Fe_2_p peak for this sample at binding energy of about 709.88 eV is associated with Fe0 [44]. For Binding energies of A4 at about 733.48 and 724.08 eV corresponds to Fe^3+^ and Fe^2+^ respectively. As reported by Li et al. that the position of the Cu_2_p band relative to the Fermi level is lower than that of the Fe_2_p band [43] and therefore, would not influence strongly its electronic structure. XPS analysis confirmed that co-sputtered Fe-Cu thin films are more stable then layer deposited counterparts.

**Figure 11:**
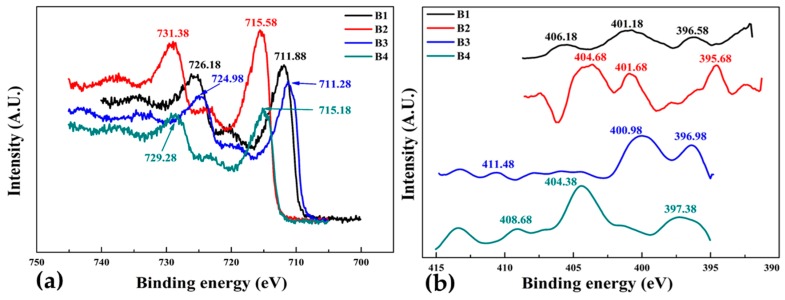
X-ray photoelectron spectra of group 2, FeN thin films deposited at different nitrogen flow rates. (**a**) Fe_2_P and (**b**) N1s.

Figure 11a,b illustrates the XPS Fe_2_p and N1s spectra for FeN thin film samples B1, B2, B3 and B4 deposited under varying nitrogen flow rates given in Table 1. There are two prominent Fe_2_p peaks (b) for all samples at binding energies ranging from 711.28 and 731.38 eV, are associated with Fe2^+^ and Fe3^+^ [45] and confirms the occurrence of two chemical bonding state in the iron nitride thin films. XPS N1s spectra, Figure 11b for all four samples show three components at binding energies 395–411 eV corresponding to a nitride layer. Sample B4 has the lowest Fe_2_p and highest N1s peak intensities than other samples owing to the presence of Fe_4_N and Fe_8_N phases and different environments nearby nitrogen atoms in the film. From the XPS N1s spectra it can also be seen that the intensities of the N1s peaks decrease sharply as flow rate decreases for samples B1 to B4 decreases. This is caused by the decrease of nitrogen content in the films by decreasing the nitrogen flow rate from 40 sccm to 10 sccm during deposition.

**Figure 12:**
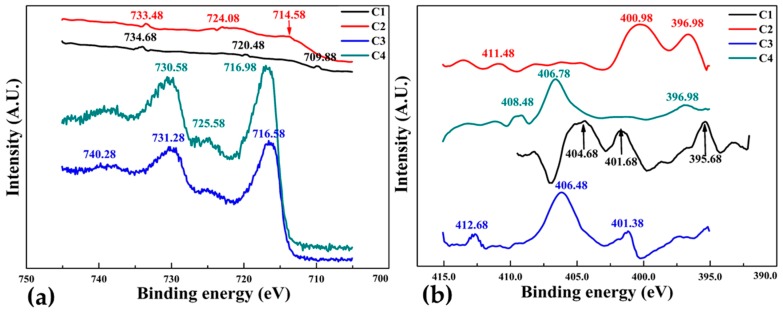
X-ray photoelectron spectra of group 3, FeN thin films deposited at different deposition time spans. (**a**) Fe_2_P and (**b**) N1s.

Figure 12a,b illustrates the XPS Fe2p and N1s spectra for FeN thin film samples C1, C2, C3 and C4 deposited with increasing deposition time spans as given in Table 1. There are three prominent peaks in both spectra of all samples which means the presence of three different chemical bonding state in the iron nitride thin films, this is also evident from the different binding energies of the samples. There is a distinguished component of the Fe_2_p peak (Figure 12a for sample C1 at binding energy of about 709.0 eV is associated with Fe0 [44], while the peaks at the higher energy in XPS Fe_2_p spectra for sample C2, C3 and C4 are related to Fe^2+^ and Fe^3+^. The maximum binding energy increases steadily for samples C1 to C3 from 733 eV to 740 eV with the increasing thickness of thin films from 74 nm to 94 nm, but it decreased to 730 eV in sample C4 which is 184 nm thick. This is because of the formation of numerous magnetic domains due to film thickness [46], leads to high Hc and the development of magnetic isotropy. Figure 12b shows the N1s spectra, binding energy at about 412 eV of sample C3 is higher than other samples and the intensity of sample C2 is greatest. It can also be seen that for samples C3 and C4 the intensities of the N1s peaks decrease abruptly as nitrogen content decreases. The XPS spectra suggests that films with thickness in the range of 94 nm are more stable than 184 nm.

**Figure 13:**
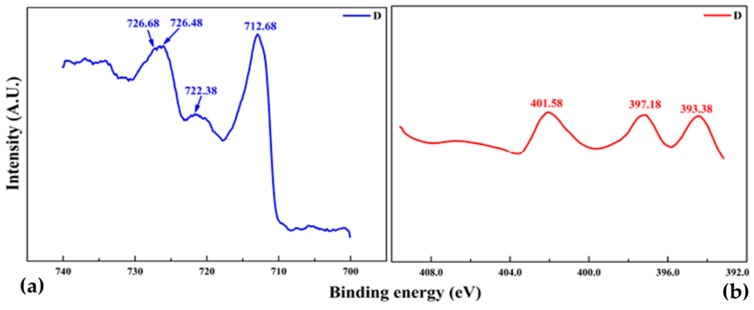
X-ray photoelectron spectra of FeN thin film sputtered on both sides. (**a**) Fe_2_P and (**b**) N1s.

Iron nitride thin film deposited on both sides on a polymer substrate of Sample D mainly consist of two phases, Fe_3_N and Fe_4_N depicted in XRD, Figure 13a,b illustrates the XPS Fe2p and N1s spectra. The two prominent Fe2p peaks Figure 13a at binding energies of about 712.68 and 722.38 eV are associated with Fe^2+^, while closely located peaks at about 726.68 and 726.48 eV are related to Fe^3+^. While XPS N1s spectra for sample D deposited at 10 sccm nitrogen flow exhibit three components which are in the range of 393–401 eV, as shown in Figure 13b. All these N1s peaks correspond to a nitride layer. The calculated values of N/Fe atomic ratio are 0.30 and 0.34 and are close to the ideal value for the phase of Fe_4_N and Fe_3_N respectively. This confirms the XRD results in principal, the error in all the peak position is expected to be 0.05 eV.

### 3.4. Surface Free Energy

**Figure 14:**
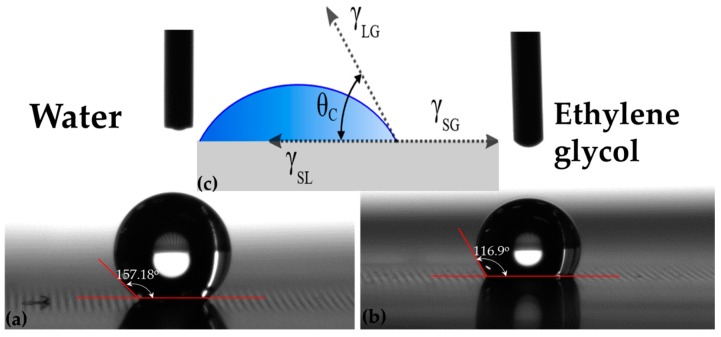
Digital images of contact between testing fluids and FeN thin film at room temperature. (**a**) Contact angle with water; (**b**) Contact angle with Ethylene glycol; and (**c**) Inset is a sketch of the sessile drop technique where θ_C_ is the contact angle, and γ_LG_, γ_SG_, γ_SL_ represent the liquid-gas, solid-gas, and solid-liquid interfaces, respectively.

When a surface is formed, the particles at the surface loose the equilibrium, which they primarily possessed in the bulk and extra forces are essential to preserve the molecules at the surface in the stable state. The uneven forces for the molecules at the surface lead to an extra energy at the surface, and this additional energy at the surface is known as surface energy. A break in the physicochemical uniformity of the bulk instigates the surface free energy and depends on the contact angle between surface of thin film and testing liquid. Contact angle values for two different testing liquids, water and ethylene glycol were measured from the digital pictures of the droplets on the as deposited thin films. Contact angles, which are a quantitative measure of wetting of a film by a liquid for a representative sample, are shown in Figure 14.

The key principle of the method is that by placing a droplet of liquid with a known surface energy and shape of the drop, specifically the contact angle are the parameters which can be used to determine the surface energy of the solid sample. Wetting a solid surface by a liquid is a surface phenomenon in which the liquid spreads on the surface and be likely to cover it. Wetting of solid surfaces can be categorized into two types: nonreactive wetting, in which a liquid spreads on a substrate with no chemical reaction or absorption, and reactive wetting by chemical reactions between spreading liquid and substrate material. The two different extreme equilibrium regimes may be described by the value of contact angle as: complete wetting (θ_C_ = 0), or absolute non-wetting (θ_C_ ≥ 180°). When the contact angle is a finite value between zero and 180°, the surface is then partially wetted by the liquid. Acid-Base, OWRK (Owens, Wendt, Rabel and Kaelble)/Fowkes, and Equation of State approaches were followed to calculate the surface free energy (SFE) for iron and iron nitride thin films. The total surface energy, both for a solid and a liquid is simply the sum of polar (γ^p^) and dispersive (γ^d^) components interaction as described by the following equation.

(3) $$\gamma_{L}\;\left(1+\cos\theta \right)=2\left\{\sqrt{\gamma_{L\;}^{d}\gamma_{S}^{d}}+\sqrt{\gamma_{L\;}^{p}\gamma_{S}^{p}}\right\}$$

$$\gamma_{L}=\mathrm{Surface}\;\mathrm{free}\;\mathrm{Energy}$$

Table 2 shows the measured contact angles and calculated surface free energy [13,47] for thin films on polymer substrate. The surface energy (SE) of Ag printed PET substrate, sample A1 was calculated to 39.59 mJ/m^2^ in the as received state. After sputter deposition of pure iron thin film on polymer substrate the SE of sample A2 increased to 51.88 mJ/m^2^. When pure iron thin film was doped with pure copper as another layer on top, the SE of sample A3 decreased to 18.41 mJ/m^2^. Sample A4 was also doped with pure Cu using the co-sputtering technique resulting in an increased SE value of 43.34 mJ/m^2^. This may be due to the presence of pure Fe and Cu particles on the surface of thin film as shown in XRD data, Figure 2 and suggests that co-sputtering is a more effective technique as compared to multi layers. Samples A1, A2 and A3 are unwettable by water and partially wettable by ethylene glycol because the viscosity of ethylene glycol which is a polar liquid is 25 times more than the viscosity of water hence decrease in the contact angle was observed.

With the decrease of nitrogen content from sample B1 to B3, SE increased from 46 to 155 mJ/m^2^, all samples in group 2 are unwettable by water and partially wettable by ethylene glycol at room temperature. Hence Iron nitride thin films of samples B1–B4 are hydrophobic surfaces for practical use. SE of iron nitride sample C1 is 155 mJ/m^2^ and is highest in group 3. With the increase in film thickness from 74 nm to 184 nm of sample C4 the SE decreases sharply and was calculated as 125.76 mJ/m^2^. All samples in group 3 (C1–C4) are wettable by ethylene glycol and are hydrophobic in nature. Double sided sputtered deposited FeN thin film, sample D, showed higher SE on one side then the other as shown in Table 2, Ds has SE equals to 104.37 mJ/m^2^. This is because of lower polar component and higher dispersive component along with decreased contact angle with ethylene glycol as compared to water. It is evident from our results that surface energy decreases as the θc of glycol increases and makes the thin film surface from polar to nonpolar and vice versa with water.

### 3.5. Power Absorption (Db Loss Factor)

**Figure 15:**
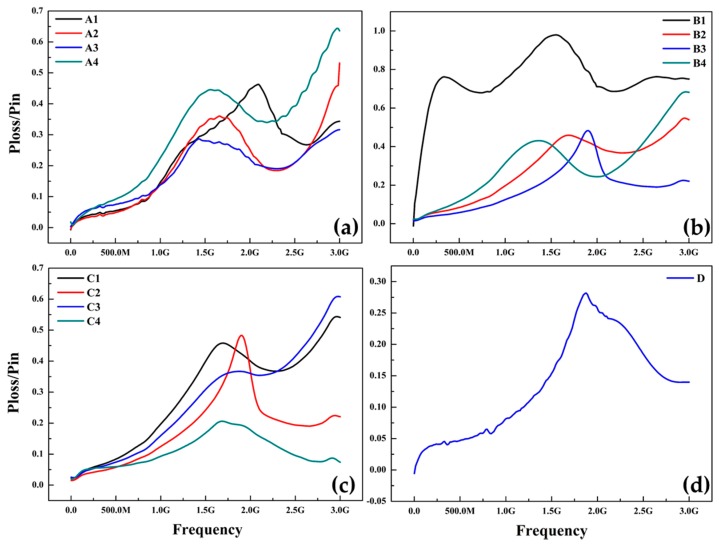
Power absorption (P_loss_/P_in_) characteristics of thin films on PET substrate (**a**) Iron and Cu dopped; (**b**) FeN with varying N2 flow rates; (**c**) FeN after different deposition time spans; (**d**) FeN deposited on both sides of PET.

Development of thin films with enhanced resonance absorption properties and high electrical resistance are needed to suppress undesirable high-frequency electromagnetic noise in highly integrated electronic devices working in the gigahertz bluetooth range. Power absorption characteristics were explained as a function of frequency with copper doping and iron nitride phase evolution in Figure 15. Shielding effectiveness or power absorption is the ratio of power loss to input calculated from the measured values of S11 and S21 which is given by Equation (4) as follows.

P_loss_/P_in_ = 1 − {(S_11_)^2^ + (S_21_)^2^} (4)

Electromagnetic interference (EMI) shielding of high frequency radio waves in electronic devices has become a serious distress in modern society in concurrence with the demand for RF radiation sources. Along with the higher saturation magnetization, increased values of magnetic permeability, and magnetic loss can be obtained in gigahertz frequencies above Snoek’s limit [48,49] and the EMI shielding effectiveness according to Schelkunoff theory (SE = A + R + B) is the total sum of absorbed, reflected and internal reflected wastage [50]. Figure 15a shows the SE of sample A1 is 43% and A2 is 35% and with the deposition of Cu layer SE of sample A3 decreased to 26% due to nonmagnetic nature of Cu with obviously large reflection.

SE of sample A4 reaches to a maximum of 65% suggesting that co-sputtering is more effective than layer deposition of Cu on Fe thin film, this is in agreement with XPS and free energy results. Figure 15b shows the SE of sample B1 is around 88% at 1.5 G and decreased to a more stable 60% power absorption between 2 and 3 GHz frequency ranges. In the lower frequency region (1.5–2 GHz), power absorption increases with both frequency and nitrogen flow rate. In the region of 2–3 GHz, power absorption is saturated as high as 88%. As with more decreased nitrogen flow rates, samples B2, B3 and B4 shows a resonance frequency 1.4–2 GHz and at 10 sccm of N2 sample B4 exhibits a maximum shielding effect of 62%. At varying sputtering deposition time spans, samples C1–C4 shows a resonance frequency at 1.9 GHz and sample C3 (94 nm thick) reveals highest SE of 66% at 3 Ghz. These results confirm the XPS and VSM analysis, the high permeability in GHz frequency and low energy loss can be accomplished by adjusting the component and phase evolution of the thin films. Where (S_11_)^2^ and (S_21_)^2^ denoted the power conducted from port 1 to port 2 and vice versa, respectively. Figure 15c shows the variations in the S_12_ parameter as a function of frequency for altered thicknesses of thin films. The S_12_ values amplified with increases in film thickness in the Bluetooth frequency range of 1.50 to 3.00 GHz. Figure 15d shows the low or negligible SE of double sided deposited FeN film, owing to the thin thickness of film (26 nm). For samples C1–C3, in the frequency region below 2 GHz, power absorption increases with both frequency and film thickness. In the region of 2.5–3 GHz, power absorption B1 and C3 is saturated as high as 88% and 65% respectively making them a potential candidate for EMI shielding applications. Power absorption/loss of those magnetic thin films is due to many factors such as material loss, frequency, and film thickness. Likewise, the resonance frequency stretching from 1.5 to 3 GHz can be tuned by controlling film thickness [51]. This indicates that EMI shielding by soft magnetic thin films is promising and the performance can be extended to more miniaturized and integrated components.

### 3.6. Structure and Morphology (TEM)

**Figure 16:**
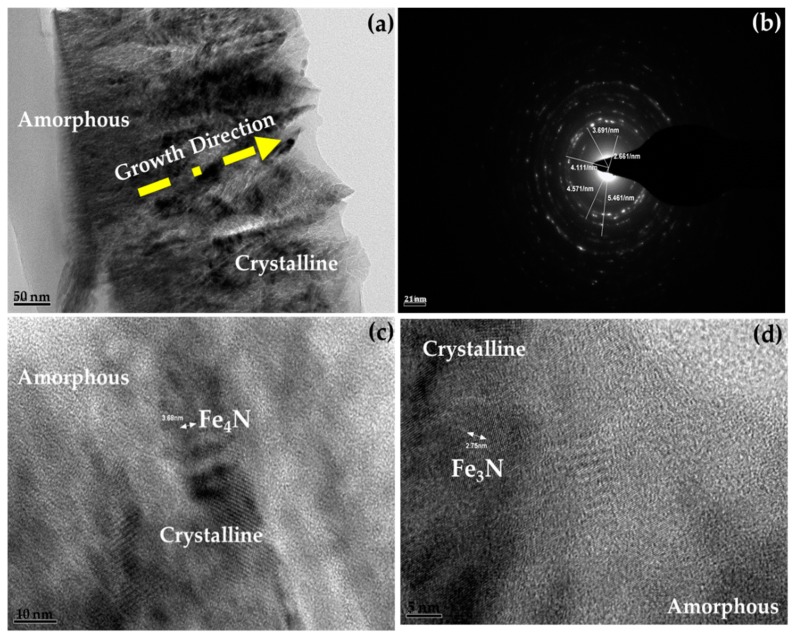
TEM micrographs of the FeN thin film deposited for 15 min on both sides at 10 sccm nitrogen flow rate (**a**) TEM image; (**b**) SAED pattern and (**c**), (**d**) HRTEM image.

Transmission Electron Microscopy (TEM) is the best technique for investigation of the size, shape and growth direction of thin films [2]. To investigate the structure and morphology of thin films more evidently, we performed (TEM) observation. Figure 16 shows micrographs and SAED pattern of double sided iron nitride thin film deposited on PET substrate at room temperature (sample D).

The TEM images shown in Figure 16a indicate that irregular cubic γ′-Fe_4_N and ε-Fe_3_N nano particles oriented in preferred directions, these two phases uniformly disperse in the amorphous iron nitride matrix according to the standard powder diffraction files JCPDS#77-2006 and JCPDS#73-2101 respectively. The nano-particles are detached from each other by 2.5–3.5 nm and have an average diameter of 5–20 nm. Figure 15b exhibits the diffraction rings from (110), (111) and (012) planes of cubic γ′-Fe_4_N along with (200) and (220) planes of ε-Fe_3_N. No other diffraction rings are present corresponding to crystalline iron nitride indicating that iron nitride matrix is amorphous. Amorphous regions in thin films are featureless having no grain boundaries, which frequently results in a discharge of intrinsic stresses, a decrease in magnetic anisotropy, and a smoother surface or interface. TEM Figure 16c shows the high resolution image (HRTEM) of crystalline Fe_4_N grains with the (111) lattice spacing of 3.68 nm and a thin amorphous layer with loose structure can be detected around the iron nitride nanoparticles. While crystallized Fe_3_N grains are evident in Figure 16d with lattice spacing of 2.54 nm which confirms that film thickness can significantly affect the structure of thin films. That if more nitrogen atoms insert into the iron lattice it can easily convert the ε-Fe_3_N into γ′-Fe_4_N. As can be seen that all thin films have crystalline structure deposited at room temperature, which is not in agreement as reported by Rissanen et al. [52], or this maybe an added advantage of using polymer substrate.

## 4. Conclusions

Static magnetic properties confirmed that the dominant phase, changes steadily from the ferromagnetic α-Fe (N) to the paramagnetic ξ-Fe_2_N with the increase of nitrogen flow rates due to ordering of the nitrogen atoms. Crystalline iron-nitride thin films on PET substrates can be synthesized using reactive magnetron sputtering by keeping the substrate at room temperature. Thickness of samples increased with the increase of deposition time span and nitrogen flow rates. A unique Iron nitride film deposited on both sides reveals co-existence of two ferromagnetic phases, γ′-Fe_4_N and ε-Fe_3_N and shows negligible magnetic anisotropy hence can be considered a potential candidate for double sided transparent display panels. XPS analysis and Contact angle calculations confirmed that co-sputtered Fe-Cu thin films are more stable then layer deposited counterparts. Thick films (184 nm) are less stable because of weak energy between atoms. Surface energy decreases as the contact angle of glycol increases and makes the surface of thin film from polar to nonpolar and vice versa with water. Iron nitride film deposited in 20 sccm N_2_ flow achieved highest surface free energy of 155.217 mJ/m^2^ and acceptable power absorption of 65% in blue tooth frequency range. Which suggests that the films have prospective uses in high frequency field.

## Figures and Tables

**Table 1:** Variable processing parameter sample ID’s during deposition.

Sample Groups ID		Target	N_2_ Flow Rate (SCCM)	Ar Flow Rate (SCCM)	DC Power (W)	Sputtering Time (min)	Film Thickness (nm)
**Group 1**	**A1**	Pure PET					
	**A2**	Fe	---	60	115	15	33
	**A3**	Fe + Cu	---	60	115	10 + 5	38
	**A4**	Fe & Cu	---	60	115	15	34
**Group 2**	**B1**	Fe	**40**	48	115	20	73
	**B2**	Fe	**25**	48	115	20	74
	**B3**	Fe	**20**	48	115	20	79
	**B4**	Fe	**10**	48	115	20	80
**Group 3**	**C1**	Fe	10	48	115	**20**	74
	**C2**	Fe	10	48	115	**30**	79
	**C3**	Fe	10	48	115	**45**	94
	**C4**	Fe	10	48	115	**55**	184
**Group 4**	**D**	Fe	10	50	115	15 both sides	22.3 (first side) 13.6 (second side)

**Table 2:** Surface free energy calculated from measured contact angle data of thin films deposited on PET substrate.

Sample	Contact Angle (θc)		γ_s_^p^	γ_s_^d^	Surface Free Energy (mJ/m^2^)
	Water	Ethylene Glycol			
**A1**	61.87	40.87	25.2111	14.3795	39.59
**A2**	108.03	67.03	1.2806	50.6077	51.8838
**A3**	129.85	102.97	0.8296	17.5868	18.4164
**A4**	118.2	80.47	2.2575	41.0896	43.3470
**B1**	123.37	83.9	3.8862	42.5508	46.4371
**B2**	135.3	63.96	33.0792	122.1382	155.2174
**B3**	142.3	69.7	36.6031	118.4033	155.0063
**B4**	119.1	78.6	3.4172	46.6760	50.0932
**C1**	135.3	63.96	33.0792	122.1382	155.2174
**C2**	142.3	69.7	36.6031	118.4033	155.0063
**C3**	130.06	73.8	16.3404	80.3071	96.6474
**C4**	133.13	69	24.6273	101.1358	125.7630
**Da**	157.18	116.9	6.4037	20.0773	26.4810
**Db**	137.57	78	21.3612	83.0094	104.3705
